# Supplementary figures and images for: A voice-based algorithm can predict type 2 diabetes status in USA adults: Findings from the Colive Voice study
Source: PLOS Digit Health. 2024 Dec 19;3(12):e0000679. doi: 10.1371/journal.pdig.0000679 (PMC11658629; doi:10.1371/journal.pdig.0000679)

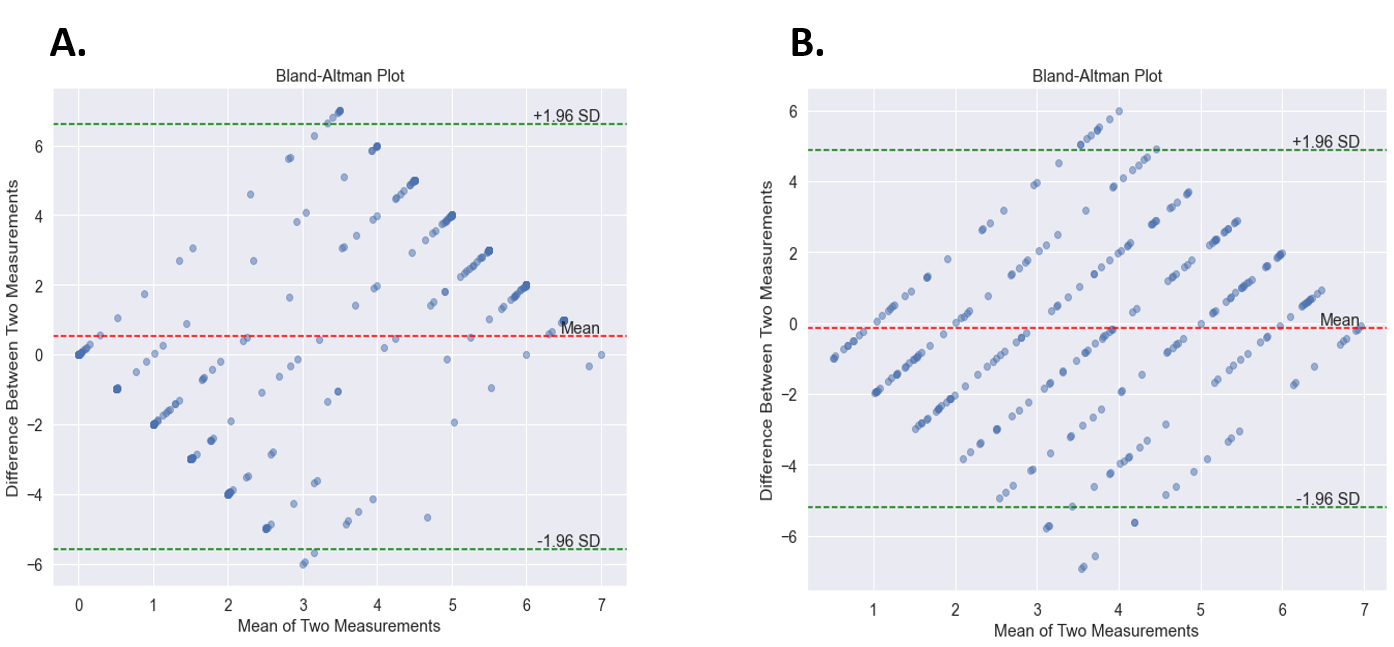

Supplement: S1 Fig — Bland-Altman plot showing the agreement between the voice-based algorithms’ predicted probability and the ADA risk score for both gender groups (A: Female group, B: Male group). Note: The predicted probability was scaled by a factor of 7 for harmonization. (TIF) [file pdig.0000679.s001.tif]
