# Supplementary material for: A voice-based algorithm can predict type 2 diabetes status in USA adults: Findings from the Colive Voice study
Source: PLOS Digit Health. 2024 Dec 19;3(12):e0000679. doi: 10.1371/journal.pdig.0000679 (PMC11658629; doi:10.1371/journal.pdig.0000679)
Supplement: Attachment — Submitted filename: Rebuttal letter_PLOS digital health.pdf [file pdig.0000679.s002.pdf]

LUXEMBOURG INSTITUTE OF HEALTH

To: Leo Anthony Celi, Editor-in-Chief, PLOS Digital Health,

Dear Editor-in-Chief,

Thank you for considering our manuscript entitled "**A voice-based algorithm can predict type 2 diabetes status in USA adults: Findings from the Colive Voice study"** for publication in **PLOS Digital Health**. We are grateful to the Reviewers for their positive and constructive remarks.

We have now taken all the comments into account and the manuscript has substantially been improved. We believe it will be of great interest to the readership of PLOS Digital Health.

Please find below a point-by-point response to the Reviewers' comments.

We thank you for your consideration of this manuscript and we look forward to hearing from you.

Sincerely yours

Corresponding Author

Guy Fagherazzi, MSc, PhD

Director, Department of Precision Health

Head, Deep Digital Phenotyping Research Unit

Luxembourg Institute of Health

1A-B, rue Thomas Edison, L-1445 Strassen, Luxembourg

Tel: +352 26970-457 / Fax: +352 26970-719

Email: guy.fagherazzi@lih.lu

LUXEMBOURG
INSTITUTE
OF HEALTH

Reviewer #1: The study explores using voice-based algorithms to predict T2D status in US adults, aiming to develop a non-invasive, scalable screening method. The authors analyzed text recordings from 607 Colive Voice study participants and used hybrid BYOL-S/CvT embeddings to create gender-specific algorithms for T2D prediction. The algorithms were evaluated using cross-validation, and their performance was stratified by age, BMI, and hypertension, and compared to the ADA score for T2D risk assessment.

1. The study did not provide detailed information on the recruitment process and inclusion/exclusion criteria for participants. There may be potential selection bias if certain groups of individuals were more likely to participate in the study.

We appreciate the Reviewer's concern regarding the recruitment process and potential selection bias. To address this, we now provide a comprehensive overview of the recruitment process and inclusion/exclusion criteria used in our study.

"To ensure diversity, Colive Voice collects voice recordings from volunteers above the age of 15 years, regardless of their health status and conditions, in English, French, German, and Spanish globally. Each participant contributes with standardized vocal tasks which are then annotated with clinical and demographic data."

Here are the key details of our recruitment and data handling, all included in our manuscript:

Study population: Colive Voice is a worldwide, multilingual research program that includes participants from diverse backgrounds and health conditions. The inclusion criterion is being above 15 years of age, with no exclusion based on health status.

Control group selection: Individuals without endocrine diseases, including diabetes, were selected randomly from the pool of US participants to create a control group that matched the size of the group of participants with T2D. This random sampling helps mitigate selection bias, ensures balanced group sizes for effective training of our machine learning algorithms, and limits the challenges related to imbalanced datasets.

Ethics statement: The study was approved by the National Research Ethics Committee of Luxembourg (study number 202103/01) and registered on ClinicalTrials.gov (NCT04848623). All participants provided informed consent.

Data collected: Participants complete a comprehensive questionnaire covering demographic characteristics, lifestyle habits, anthropometric data, symptoms, drug use, and history of chronic diseases. For diabetes-specific data, the study gathers information on diagnosis, type, duration, treatment categories, and HbA1c levels.

2. The study included participants with diverse T2D durations but did not specifically target early-stage T2D or prediabetes cases.

We appreciate the Reviewer's observation. Indeed, our study included participants with varying durations of T2D. The primary aim of our research was to explore the potential of voice analysis to differentiate between individuals with and without T2D. As stated in our manuscript: "Our goal was to explore the possibility of using a rapid, user-friendly voice recording as a T2D status predictor."

In our dataset, the diabetes duration distribution is as follows:

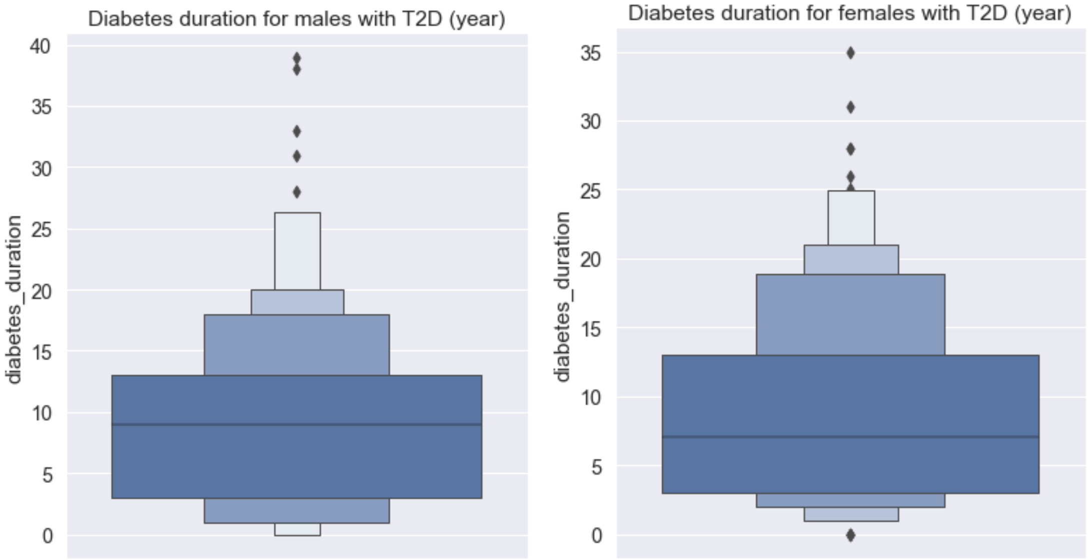

This graph shows the high variability in the profiles included in our analysis. With an average diabetes duration of about 9 years, we studied both recent diabetes (ie. <5 years since diagnosis, where diabetes complications are rare) and older cases (> 15 years of diabetes duration, where diabetes complications are more frequent).

Additionally, we have now acknowledged the need for further research to refine and validate this approach specifically for early-stage T2D and prediabetes detection. Our recommendations emphasize: "To robustly establish and reinforce the performance of a future screening tool in predicting T2D, a more diverse and large dataset is needed, while specifically targeting early-stage T2D and prediabetes cases."

We recognize the importance of targeting these specific cases and plan to address this in future studies to enhance the applicability of voice analysis as a first-line comprehensive screening tool.

3. Due to data constraints, physical activity levels and family history of diabetes were not available and were assigned a default value of zero for all participants. This may introduce less variability in the ADA scores and potential misclassification.

We acknowledge the Reviewer's concern regarding potential misclassification due to assigning default values for physical activity and family history of diabetes. However, the ADA diabetes risk score is predominantly influenced by age and BMI, which are accurately available in our study.

LUXEMBOURG INSTITUTE OF HEALTH

The ADA diabetes risk test includes the following components and their respective points:

Component	Categories	Points
Age	<40 years	0 points
	40-49 years	1 point
	50-59 years	2 points
	≥60 years	3 points
Gender	Female	0 points
	Male	1 point
Gestational Diabetes Mellitus (GDM)	No	0 points
	Yes	1 point
Family history of diabetes	No	0 points
	Yes	1 point
High blood pressure	No	0 points
	Yes	1 point
Physical activity	Yes	0 points
	No	1 point
Obesity (based on BMI)	BMI <25	0 points
	BMI 25-29.9	1 point
	BMI ≥30	2 points

As mentioned in the article "…the impact of this limitation is somewhat limited since the ADA score is primarily driven by age and BMI, which are available in our study."

Given this scoring system, age and BMI contribute significantly to the total score (up to 5 points out of 11 (45% of the total score). The impact of physical activity and family history, together contribute a maximum of 2 points less than 20% of the maximum score), is less substantial. Thus, the accurate data for age and BMI ensures the majority of the score's variability is captured.

Future work will aim to collect comprehensive data, including physical activity and family history, to further enhance the accuracy of our assessments.

4. While the study performed additional analyses to identify important subgroups and compared the influence of key demographic and health parameters, the interpretability and explainability of the algorithms could be further improved.

We appreciate the Reviewer's feedback on the interpretability and explainability of our algorithms. This was indeed a significant focus of our study, addressed through our performance stratification analyses.

Our approach involved:

Performance stratification: Recognizing that embeddings and pre-trained algorithms often function as black boxes, we emphasized analyzing clinical data to identify cofactors affecting voice and key risk factors and symptoms of T2D. This stratification aimed to reveal how different demographic and health parameters influence the algorithm's performance.

Focus on clinical relevance: By comparing the influence of specific cofactors, such as age, BMI, hypertension, and other health conditions, we aimed to enhance the clinical relevance and interpretability of our findings. This analysis helps in understanding which subgroups benefit most from voice-based T2D detection and why.

Future work: We fully agree with the need to further improve the interpretability and explainability of such models. Future work should extend the dataset and continue to refine the methods to achieve this goal. Future work should focus on increasing the robustness, interpretability, and overall, trustworthiness of voice-based algorithms for diabetes.

We believe these steps will significantly enhance the clarity and clinical applicability of our voice-based T2D detection algorithms, ensuring they are both effective and understandable for broader use.

5. The study did not account for potential confounding factors that may influence voice characteristics, such as smoking, alcohol consumption, or other underlying health conditions.

We appreciate the Reviewer's concern. Our study did account for potential confounding factors through performance stratification.

Performance stratification: We specifically analyzed the influence of various cofactors, including smoking, age, BMI, and other underlying health conditions, on the performance of our voice-based T2D detection algorithms. By examining conditions such as hypertension, migraine, diagnosed depression, stress, and fatigue, we assessed how these factors might affect voice characteristics and the algorithm's predictive accuracy.

Focus on language and gender: To ensure a comprehensive analysis and minimize biases, we focused on English-speaking participants and treated each gender separately. Recognizing the significant differences in voice characteristics and health profiles between genders, this separation allowed for more accurate and relevant findings.

By incorporating these stratifications focusing on a single language and separating genders, we aimed to mitigate the influence of confounding factors and enhance the robustness of our findings.

6. The study used cross-sectional data, which limits the ability to establish causal relationships between voice characteristics and T2D status.

We appreciate the Reviewer's insight regarding the limitations of using cross-sectional data. We acknowledge that cross-sectional data inherently limits our ability to establish causal relationships between voice characteristics and T2D status. Our study aimed primarily to explore the potential of voice analysis as a predictor of T2D status, rather than to establish causality.

However, to address this limitation and strengthen our findings, we have the following recommendations/plans for future research:

Longitudinal studies: Conduct longitudinal studies that follow participants over time. This will help to better understand how changes in voice characteristics correlate with the development and progression of T2D. Additionally, it will provide insights into the main clinical diabetes-related parameters, such as glycemic control and diabetes-related complications, and establish causal relationships. This is now added to our discussion: "Additionally, conducting longitudinal studies will help to better understand how changes in voice characteristics correlate with the development and progression of T2D. This approach will provide insights into the main clinical diabetes-related parameters, such as glycemic control and diabetes-related complications, and help establish causal relationships."

Expanded data collection: Expand our dataset to include repeated measurements and track participants' health status over an extended period. This approach will provide a more comprehensive understanding of how voice characteristics evolve with T2D.

Enhanced analytical methods: Future research will incorporate advanced analytical methods, such as time-series analysis and causal inference techniques, to better assess the potential causal links between voice features and T2D status.

By taking these steps, we hope to build on our initial findings and provide more robust evidence for the use of voice analysis in T2D detection and monitoring.

7. The study relied on a sample of English speakers only, which may limit the generalizability of the findings to other languages and populations.

We appreciate the Reviewer's concern regarding the generalizability of our findings. Our decision to focus on a single population of English speakers was intended to reduce significant biases that may arise from cultural and linguistic differences. Different cultures, languages, and countries have varying settings, risk factors, and health outcomes, which could affect the voice characteristics and T2D status.

To address this, we emphasize the following:

Minimizing bias: By limiting our study to English speakers, we aimed to control for language and cultural variables that could introduce bias and affect the reliability of our findings.

Future research: Extending this work to broader populations and languages is crucial. As highlighted in our discussion, "It is also important to generalize this research across different populations, with diverse backgrounds and languages. Expanding datasets will allow a deeper examination of nuanced factors, comorbidities, and their interactions affecting voice-based screening tools in predicting T2D."

We acknowledge the importance of validating our findings across diverse populations and languages to enhance the generalizability and applicability of our voice-based T2D detection algorithms. Future studies should focus on this aspect to ensure broader relevance and impact.

8. The study did not include an external validation dataset to assess the performance of the developed algorithms.

We acknowledge the Reviewer's concern about the lack of an external validation dataset. While our study demonstrated promising results using internal cross-validation, we recognize the importance of external validation to assess the generalizability of our algorithms. We plan to address this in future research by incorporating external datasets from diverse populations and settings to validate and further refine our voice-based T2D detection algorithms. This step is crucial to ensure robustness and applicability in broader, real-world contexts.

9. Although the study used a larger sample size compared to previous studies, the sample size may still be insufficient to capture the full spectrum of voice variations associated with T2D.

We fully agree with this observation. While our study utilized a larger sample size compared to previous research, an even more extensive dataset is required to capture the full spectrum of voice variations associated with T2D, particularly early-stage diabetes and prediabetes. Future work will focus on expanding the dataset to include these critical stages, ensuring a comprehensive analysis of voice characteristics across the entire spectrum of T2D: "To robustly establish and reinforce the performance of a future screening tool in predicting T2D, a more diverse and large dataset is needed, while specifically targeting early-stage T2D and prediabetes cases.".

10. While the study compared the performance of the voice-based algorithms with the ADA risk score, it did not compare them with other established screening methods, such as fasting blood glucose or HbA1c tests.

We appreciate the Reviewer's suggestion. Our study focused on comparing the voice-based algorithms with the ADA risk score, which assesses risk rather than serving as a primary screening tool. This distinction is important as we aimed to

explore the potential of voice as a non-invasive risk assessment tool that may serve as a first-line screening tool. However, we recognize the importance of comparing it with established screening methods like fasting blood glucose or HbA1c tests. This will be an area of further investigation.

LUXEMBOURG INSTITUTE OF HEALTH

Reviewer #2: The manuscript by Abir Elbeji et al developed a novel screening tool for diagnosing type 2 diabetes mellitus by building a voice-based machine-learning algorithm.

Although the data presented is clearly interesting, there are several issues that will require further clarification prior to publication:

1. The authors are advised to clearly describe how type 2 DM are diagnosed and defined. What is the diagnositc criteria for Type2 DM in this study?

The diagnosis and definition of Type 2 DM in this study were based on self-reported information provided by participants. Specifically, participants reported their diabetes diagnosis, type, duration since diagnosis, and treatment categories through a comprehensive questionnaire.

Questions on Colive Voice were as folllows:
- Have you ever been diagnosed with one or several of the following diseases?
If diabetes is among them:
- What type of diabetes do you have?
- How old were you when you were diagnosed with diabetes?
- Do you use tablets to treat your diabetes?
- Do you use insulin to treat your diabetes?
- What is your most recent HbA1c result (%)?
- Do you use a continuous or flash glucose monitoring device? etc … .

2. The authors are requested to calculate the AUC by diagnosisng/evaluating participants with ADA risk scores.

   The authors are also requested to compare the AUC above with that of the develpped algorithm.

Thank you for your valuable input. In response to your request, we have performed the requested analyses and included them in the revised manuscript. Specifically, we calculated the AUC for the ADA risk score and compared it with the performance of our developed algorithm. The results showed that the AUC for the ADA risk score was 0.72 for females and 0.71 for males. Comparatively, our voice-based algorithm achieved an AUC of 0.71 (0.07) for females and 0.75 (0.05) for males.

3. What does the numerical value in the brackets in Table 2 mean? Are they standard deviation? or standard error mean?

Thank you for raising this point. The numerical values in brackets in Table 2 represent the standard deviation. We incorporated it in the revised version of the manuscript.

4. In line 297, the authors wrote, "notable differences were observed for females across...". In addition, they also wrote in line 305 that no noticeable disparities were observed among males. The authors are requested to clarify the difference between

"notable difference in women" and "no noticeable disparities among males". The authors are advised to describe how they defined "notable/noticeable difference".

We appreciate the Reviewer's request for clarification. The terms "notable difference" and "no noticeable disparities" were used to describe the variations in algorithm performance metrics between different age groups for females and males, respectively. For females, we observed significant variations in specificity, sensitivity, and AUC between the age groups:

- **Females under 60 years: Specificity (0.65 ± 0.04), Sensitivity (0.65 ± 0.04), AUC (0.65 ± 0.03).**
- **Females 60 years and above: Specificity (0.74 ± 0.12), Sensitivity (0.74 ± 0.07), AUC (0.74 ± 0.07).**

These differences are notable because there is a clear improvement in all three performance metrics for older females, indicating a significant impact of age on the algorithm's performance in this group.

Conversely, for males, the performance metrics were relatively consistent across age groups:

- **Males under 60 years: Specificity (0.70 ± 0.04), Sensitivity (0.74 ± 0.04), AUC (0.72 ± 0.03).**
- **Males 60 years and above: Specificity (0.70 ± 0.11), Sensitivity (0.70 ± 0.10), AUC (0.70 ± 0.07).**

The lack of substantial variation in these metrics indicates that age did not significantly influence the algorithm's performance for males, thus we described it as "no noticeable disparities."

To clarify, we defined "notable differences" as variations in performance metrics (specificity, sensitivity, and AUC) that were both statistically significant and clinically relevant, reflecting a meaningful change in the algorithm's ability to detect T2D. "No noticeable disparities" indicates that the differences in performance metrics were minimal and did not reflect a significant impact of the demographic factor (age) on the algorithm's performance.

5. In line 315, the authors wrote that the presence of depression significantly influenced the algorithm's performance in woman. The authors need to show the evidence of this significance.

We appreciate the Reviewer's request for clarification. The significance of depression's influence on the algorithm's performance in women was determined using the Mann-Whitney U test This is mentioned in our Methods section: "To evaluate the statistical significance of performance differences between categories, we employed the Mann-Whitney U test. ". This statistical analysis results are now

included in Table 3 in the revised manuscript, providing the necessary evidence to support our findings.

6. In line 344, they wrote AUC score of algorithm in female T2DM as 0.72. However, I'm afraid that this might be 0.71 (I think this is an innocent mistake/mistype)

Thank you for pointing out this inconsistency. You are correct; this was a typo. The correct AUC score for the algorithm in female T2DM is indeed 0.71. We have corrected this in the revised manuscript.

Reviewer #3: The present manuscript highlights an algorithm to detect type 2 diabetes (t2d) based on multidimensional data features derived from voice recordings. I have a few comments/questions for the authors. Thank you for considering my comments.

1. Introduction: The authors say the FINDRISC (Finnish diabetes risk score) has limited detection capabilities (AUC of 76%). However, their algorithm's AUC is similar or lower (75% for males, 71 for females). Would this not be an argument to use a much simpler questionnaire to assess the risk for t2b?

Thank you for this comment. We have indeed shown comparable performances of our voice-based algorithm with the ADA risk score, which has a similar philosophy as the FINDRISC. This suggests that voice-based approaches could complement, but not replace existing diabetes screening methods. However, we are not there yet with voice research. The present work focuses on demonstrating that we can distinguish people with and without T2D using the information from their voices alone. It is the first, promising step towards a voice-based screening solution, but our model alone cannot be considered as a screening solution yet.

Our research demonstrates the potential of voice as a novel, rapid, scalable, user-friendly, and non-invasive alternative to traditional questionnaires, which could improve user engagement and accessibility.

2. Sample size: The overall sample size is N=607, reported in the abstract. However, the authors performed the analyses stratified by gender. The larger group of females is N=323, with 162 events (t2d) and 161 non-events. Thus, the effective sample size for their model is only N=161. This is a rather small study to develop a model based on 200 features after dimensionality reduction. The number of features is higher as the number of events observed; how do the authors mitigate massive overfitting?

We appreciate the Reviewer's concern regarding the sample size and potential overfitting. We would like to clarify that the effective sample size for our model is 323, as the algorithm considers both T2D and non-T2D cases. Here are the measures we implemented to mitigate overfitting:

Cross-Validation: We employed stratified 5-fold cross-validation in our analysis. This technique ensures that the model is trained and validated on different subsets of the data, which helps in assessing its generalizability and mitigating overfitting.

Dimensionality reduction: Before applying the algorithm, we performed dimensionality reduction using Principal Component Analysis (PCA) for embeddings and feature selection with SelectKBest for Opensmile features. This process reduces the number of features to a manageable level, capturing the most relevant information and minimizing the risk of overfitting.

Balanced dataset: Our dataset was balanced in terms of the number of cases with T2D and without T2D. This balance is crucial for the algorithm to learn effectively from both classes and helps prevent overfitting to one class.

Regularization techniques: The machine learning models used (Logistic Regression, SVM, and MLP) inherently include regularization parameters that help control overfitting by penalizing overly complex models.

Despite these measures, we acknowledge that larger and more diverse datasets are now needed. Future studies should include a larger sample size and validate the model across different populations to ensure its reliability and generalizability.

3. Methods: The authors state they use TRIPOD reporting guidelines; however, it was not reported whether the study had missing data or not and how missing data was handled if present.

Thank you for pointing this out. We confirm that there was no missing data in our study. We have added this information to the revised manuscript to ensure clarity and adherence to the TRIPOD reporting guidelines.

4. Methods: What was the rationale for performing the analysis separately, stratified for males and females? Why was the model not developed on all the data, and why is sex used as one of the prognostic factors along the features?

The rationale for performing the analysis separately, stratified for males and females, was to account for major gender differences in voice characteristics and to mitigate gender bias. Voice features can vary significantly between males and females due to physiological and hormonal differences, which can affect the accuracy and performance of the algorithm if not accounted for.

Gender differences in voice: Males and females have distinct vocal characteristics, such as pitch and frequency range. Stratifying the analysis helps ensure that the model performs well across both genders without being biased toward one. This approach enhances the generalizability and fairness of the algorithm.

Separate models: Developing separate models for males and females allows us to fine-tune the algorithms for the specific characteristics of each gender, improving overall predictive performance.

Sex was not used as a prognostic factor but rather as a stratification variable to ensure that the unique voice features of each gender were accurately captured and analyzed.

We have included this explanation in the revised manuscript to clarify our rationale. "We performed the analysis separately, stratified for males and females, to account for major gender differences in voice characteristics and to mitigate gender bias. Voice features can vary significantly between males and females due to physiological and hormonal differences, which can affect the accuracy and performance of the algorithm if not accounted for. By developing separate models for each gender, we were able to fine-tune the algorithms for the specific characteristics of males and

females, improving overall predictive performance and ensuring fairness and generalizability."

5. Methods: How was the number of components in the PCA determined?

The number of components in the PCA was determined using a grid search. This approach allowed us to evaluate various numbers of components and select the optimal configuration that maximized the performance of our algorithms. The grid search helps ensure that the selected number of PCA components captures the most relevant information while minimizing the risk of overfitting.

6. Methods: I was wondering about the performance of a very simple logistic model using sex, age, hypertension, and BMI as diagnostic factors. Would that be feasible as a benchmark?

We appreciate the Reviewer's suggestion. A simple logistic model using sex, age, hypertension, and BMI as diagnostic factors could be performed but would not directly serve the primary objective of this work. Indeed our study aimed to explore the potential of voice analysis as an innovative and non-invasive tool for T2D detection. While the performance of the voice-based model may currently be less than that of traditional logistic models using established risk factors, it offers unique advantages, such as ease of use, scalability, and the ability to be integrated into telehealth platforms or deployed on smartphones.

7. Discussion/Conclusion: The authors suggest the tool as a screening strategy for t2d; however, what is the optimal threshold to be used? For clinical implementation, this would require a decision curve analysis. Maybe the author could discuss this point.

We appreciate the Reviewer's insightful comment. At this stage, it is premature to determine an optimal threshold for clinical implementation. Our current work focuses on diabetes status detection rather than a comprehensive screening tool.

Early-stage diabetes and prediabetes: To truly develop a viable screening tool, we need to address early-stage diabetes and prediabetes. Future research will aim to include these groups to provide a more comprehensive understanding of the tool's effectiveness.

Decision curve analysis: While decision curve analysis will be critical for clinical implementation, we believe it is essential first to refine our model with a broader dataset, including individuals with early-stage diabetes and prediabetes.

For now, our study demonstrates the potential of using voice analysis for diabetes status detection. We plan to explore the optimal thresholds and conduct decision curve analysis in future studies as we expand our dataset and refine our model.
